# Annealing temperature effects on the size and band gap of ZnS quantum dots fabricated by co-precipitation technique without capping agent

**DOI:** 10.1038/s41598-023-37563-6

**Published:** 2023-06-26

**Authors:** Abduelwhab. B. Alwany, G. M. Youssef, O. M. Samir, Mohammed A. Algradee, Nabil A. A.Yahya, Mohamed A. Swillam, Syahrul Humaidi, R. Abd‑Shukor

**Affiliations:** 1 Physics Department, Faculty of Science, Ibb University, Ibb, Yemen; 2 Laboratory of Materials Sciences and Solar Cells, Physics Department, Faculty of Science, Ain Shams University, Abbasia, Cairo Egypt; 3 Engineering College, Aljanad University for Science and Technology, Taiz, Yemen; 4 Physics Department, Thamar University, Thamar, Yemen; 5 Department of Physics, School of Sciences and Engineering, American University in Cairo, New Cairo, 11835 Cairo Egypt; 6 Post Graduate Program (Physics), FMIPA, Universitas Sumatera Utara, Jln Bioteknologi No.1, Medan, 20155 Indonesia; 7 Department of Applied Physics, Universiti Kebangsaan Malaysia, 43600 Bangi, Selangor Malaysia

**Keywords:** Chemistry, Nanoscience and technology, Optics and photonics, Physics

## Abstract

ZnS quantum dots (QDs) were fabricated using the co-precipitation technique with no capping agent. The effects of different annealing temperatures (non-annealed, 240 °C and 340 °C for 2 h) on the structural and optical characteristics of ZnS QDs are reported. The samples were examined by XRD, TEM, PL, FTIR, and UV–Vis. An increase in annealing temperature led to an increase in the dot size and a lowering of the energy band gap (*E*_G_). The average crystallite size, *D* of ZnS was between 4.4 and 5.6 nm. The ZnS QDs showed a band gap of 3.75, 3.74 and 3.72 eV for non-annealed, 240 °C, and 340 °C annealed samples. The reflection spectra increased in the visible light and decreased in UV region with an increase in annealing temperature. This work showed that the band gap and size of ZnS QDs could be tuned by varying the annealing temperature.

## Introduction

Nanoparticles (NPs) research has been active in recent decades. The number of atoms on a nanoparticle's surface is larger than those inside the particle. The unique NPs properties are useful in many applications such as medicine, power generation, therapies, biological and electronics. Research on ZnO and other oxide nanoparticles has produced many interesting and rich physical properties which are useful in many applications. Other than ZnO, zinc sulfide (ZnS) nanoparticle is also interesting in terms of electrical and optical properties and has been studied quite extensively.

ZnS is an important compound among the family of II-VI group semiconductors with a wide energy band gap (*E*_G_). ZnS has two stable crystalline structures, zinc blende (cubic) and wurtzite (hexagonal). In both forms, the coordination geometry at Zn and S is tetrahedral. The *E*_G_ of wurtzite ZnS (3.79 eV) is larger than that of cubic (3.68 eV). Therefore, ZnS is potentially useful in many applications, particularly in electronics and optoelectronics such as optical sensors, electroluminescence devices, quantum dot-sensitized solar cells, and lasers^1–10^. ZnS has a large exciton binding energy (40 meV), high refractive index (2.28), and large Bohr exciton radius (2.51 nm), which enables it to be a strong candidate for possible dilute magnetic semiconductors-based devices^11,12^.

Moreover, it is an inexpensive and environmentally friendly material and a promising material for medical and biological sciences^13,14^, photocatalytic and antibacterial activities^15–17^. ZnS quantum dots are widely used in electrochemical applications due to their unique optical and electronic properties The quantum dots can be used as electrochemical sensors for various analytes such as heavy metals and DNA. Fluorencence of ZnS quantum dots can be quenched by the binding of target analytes, making them highly sensitive and selective sensors. For bioimaging ZnS has high photostability and a high quantum yield, suitable for imaging cells and tissues. With a high surface area and good electrical conductivity ZnS quantum dots are suitable for high-performance energy storage devices. ZnS quantum dots can be used as catalysts in various electrochemical reactions such as catalyzing reactions at the electrode surface.

Many works in the past have successfully synthesized ZnS nanorods, nanotubes, and nanowires by various methods^7^. Preparation methods, characterization and growth mechanism are hot spots in nanoscience at present. The synthesis of nanomaterials with different annealing temperatures is useful for developing high crystal quality and stability at different temperatures, which is essential for device purposes. Therefore, the synthesis of ZnS with different annealing temperatures can be a useful technique to optimize the optical and structural properties.

There are some works on the effects of different annealing temperatures on ZnS NPs. Nanoparticles (NPs) are typically used for particles in the nm size range and quantum dots (QDs) are nanoparticles characterized by the discretization of energy levels within the material. In our previous study, the ZnS QDs were synthesized by varying the molarity of sulfur^18^. It is interesting to investigate the effect of different annealing temperatures on the structure and optical properties of ZnS QDs. In this paper, we report on the properties of ZnS QDs annealed at 240 and 340 °C for 2 h. The non-annealed ZnS sample was also prepared for comparison. The samples were examined by X-ray diffraction (XRD), transmission electron microscope (TEM), photoluminescence (PL), Fourier transform infrared spectrophotometer (FTIR), and UV–Vis spectroscopy.

## Experimental details

### Preparation of ZnS QDs

Zinc sulfide QDs were synthesis by co-precipitation method using zinc acetate dihydrate (Zn(CH_3_COO)_2_ × 2H_2_O) and sodium sulfide (Na_2_S × XH_2_O). 0.90 M (3.951 g) of Zn(CH_3_COO)_2_ × 2H_2_O was dissolved in deionized water (20 ml). The solution was stirred magnetically for 25 min at room temperature (RT). The same process was used to prepare 1 M (1.56 g) of Na_2_S × XH_2_O solution which was slowly added to Zn(CH_3_COO)_2_ × 2H_2_O solution at room temperature (RT). The mixture was stirred (700 rpm) for 3 h to obtain a homogenous white mixture. The homogenous solution was kept at RT for 20 h and then filtered to get the precipitate. The precipitate was washed several times with deionized water and ethyl alcohol to eliminate any impurities. Lastly, the resultant powders were dried at 125 °C for 1 h in the oven and ground. The dried powders were divided into three parts. The first part was a non-annealed ZnS sample. The second and third ZnS powder parts were annealed at 240 and 340 °C for 2 h.

### Characterization of ZnS QDs

The structure and crystallite size of ZnS QDs were investigated using the X-ray diffraction (XRD) method with a Bruker D_8_ Discover diffractometer equipped with a Cu microfocus X-ray source (λ = 1.5406 Å) and a two-dimensional Vantec 500 detector. The scans were taken with a step of 0.020° from 2*θ* = 20° to 70°. The XRD data were analyzed by the Diffrac Eva software (Bruker).

Photoluminescence (PL) was measured at room temperature with an RF-6000 Spectro fluorophotometer by Shimadzu Scientific Instruments. The FTIR spectra were obtained using a Nicolet 6700 FTIR from 400 to 4000 cm^−1^ using the KBr pellet technique. The morphology of the ZnS QDs was determined by transmission electron microscopy (TEM) using HITACHI H-7500. The diffuse reflectance ultraviolet-vis spectra of ZnS QDs were obtained in the 200 to 900 nm range using a JASCO V-570 spectrophotometer equipped with an ISN-470 reflectance spectroscopy accessory.

## Results and discussion

### XRD analysis

**Figure 1:**
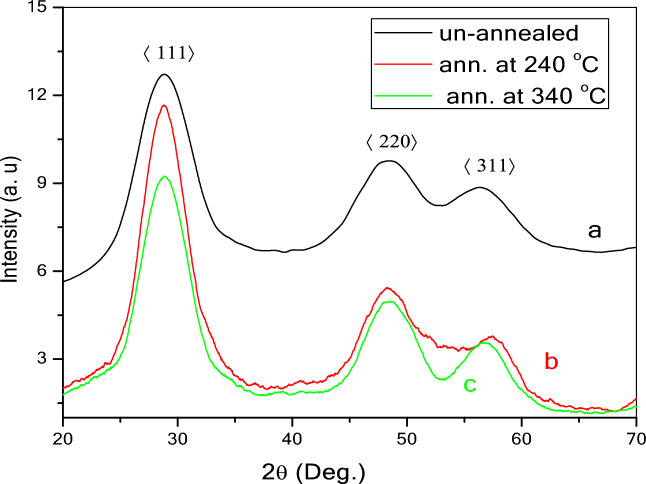
XRD patterns of (**a**) non-annealed ZnS, and ZnS annealed at (**b**) 240 and (c) 340 °C.

Figure 1 shows the XRD patterns of (a) non-annealed (b) ZnS annealed at 240 °C and (c) 340 °C. The peaks indicated the cubic structure of ZnS QDs (JCPD card number 65–9585). The cubic phase of ZnS QDs has three typical peaks with Miller indices of the most prominent peaks (111), (220) and (311). The lattice parameter, *a* and interplanar spacing *d*_*hkl*_ were determined by using^19^:

1 $$a={d}_{hkl}{\left({h}^{2}+{k}^{2}+{l}^{2}\right)}^\frac{1}{2}$$

2 $${d}_{hkl}\,\,\mathit{sin}\theta =\frac{1}{2\left(n\lambda \right)}$$

where *n* is the order of diffraction (for first-order *n* = 1), the wavelength of the X-ray radiation, ${\lambda }_{CuK\alpha }$= 1.5406 Å, and *hkl* is the Miller indices. The Debye–Scherrer equation was used to estimate the crystallite size, *D* from the broadening of the diffraction peaks^20^:

3 $$D= \frac{k\lambda }{{\beta }_{hkl}cos\theta }$$

where* θ* is the Bragg’s angle, *k* is approximated to unity and* β*_*hk*l_ is the full width at half maximum intensity (FWHM) of the XRD peaks in radians. The average strain (*ε*) and dislocation density (*δ*) of the samples were calculated using^21^:

4 $$\varepsilon = \left[\frac{{\lambda }_{cuk\alpha }}{D(cos\theta )}-{\beta }_{hkl}\right]\times \frac{1}{tan\theta }$$

5 $$\delta = \frac{1}{{D}^{2}}$$

The average lattice parameter, *a* for non-annealed and ZnS QDs annealed at 240 °C and 340 °C were 5.34 Å, 5.39 Å, and 5.35 Å, respectively (Table 1). These values were consistent with the JCPDS file no. 05–0566, (*a* = 5.4060 Å), and a previous study (*a* = 5.33 Å)^22^. The diffraction pattern of non-annealed ZnS QDs showed three peaks at 2*θ* = 28.774°, 48.329° and 56.720°. In the ZnS QDs annealed at 240 °C, three peaks were observed at 2*θ* = 28.826°, 48.429°, and 57.309°. The peaks of the sample annealed at 340 °C were observed at 2*θ* = 28.856°, 48.518°, and 56.823°. These three peaks are comparable with the standard JCPDS file no. 05–0566 which showed peaks at 28.557°, 47.513°, and 56.287°. There was a slight shift to the right of the diffraction peak relative to the standard sample in varying degrees, indicating lattice shrinkage.

**Table 1:** The 2*θ*,${\beta }_{hkl}$,* d*_*hkl*_,* d*_*stan,*_ $\varepsilon$, *δ*, *hkl* and* D* of the non-annealed and annealed ZnS QDs as determined from XRD data.

Annealed ZnS QDs	2*θ* /deg	hkl	*d*_stand_/Å	*d*_exp_/Å	*β*_hkl_/rad ± 0.0001	*a*/Å ± 0.001	*D* /nm ± 0.1	Ave. *ε*/lin^2^ m^−4^ ± 0.001	Ave. $\delta$/lin/nm^2^ ± 0.0001
Non-ann	28.774	111	3.123	3.082	0.0386	5.337	4.1	0.977	0.0509
	48.329	220	1.912	1.917	0.0443	5.422	3.8		
	56.720	311	1.633	1.586	0.0326	5.261	5.4		
Ann. at 240 °C	28.826	111	3.123	3.098	0.0364	5.365	4.4	0.933	0.0490
	48.429	220	1.912	1.909	0.0404	5.400	4.2		
	57.309	311	1.633	1.633	0.0351	5.414	5.0		
Ann. at 340 °C	28.856	111	3.123	3.093	0.0359	5.357	4.4	0.810	0.0322
	48.518	220	1.912	1.881	0.0304	5.307	5.6		
	56.823	311	1.633	1.624	0.0260	5.385	6.7		

*d*_*hkl*_ are compared with the standard of interplanar spacing (*d*_*stan.*_).

The diffraction peaks showed obvious narrowing with an increase in annealing temperature, which suggests an enhancement in the grain size and a lowering of dislocation density (*δ*) with an increase in annealing temperature. The average crystallite size, *D* of the non-annealed and annealed ZnS QDs was estimated from the FWHM of all the peaks using Eq. (3). The crystallite size of the samples lies in the range of quantum dots (i.e., *D ˂* 10 nm). *D* of the non-annealed ZnS was 4.4 nm and increased to 4.5 and 5.6 nm with an increase in annealing at 240 and 340 °C, respectively. Table 1 shows the 2*θ*,${\beta }_{hkl}$, *d*_*hkl*_,* d*_*stan,*_ $\varepsilon$, *δ*, and *hkl* of the ZnS QDs. The values of *d*_*hkl*_ obtained by XRD were in good agreement with the standard of interplanar spacing (*d*_*stan.*_) in the JCPDS file no. 05–0566.

### TEM studies

**Figure 2:**
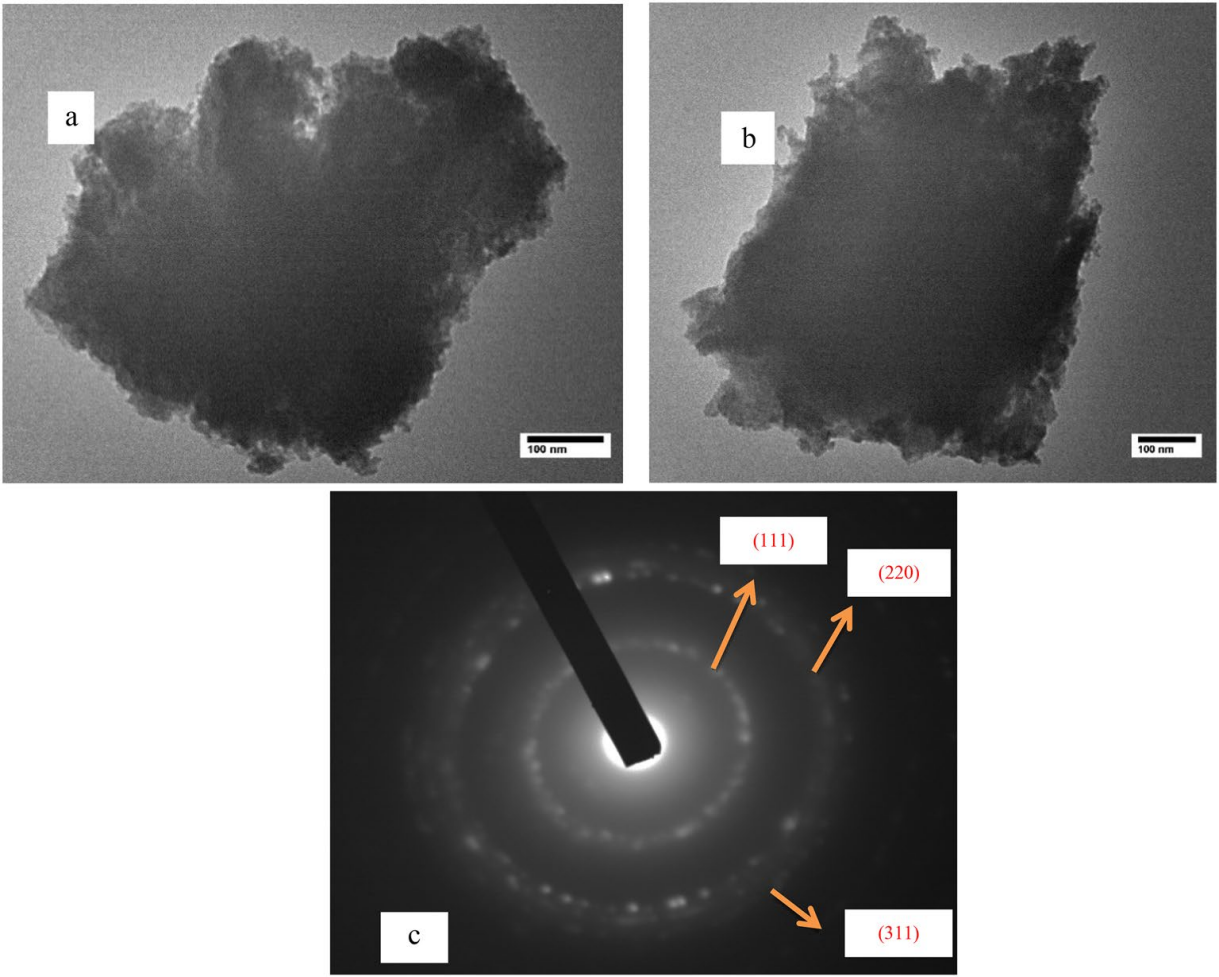
(**a**) TEM image of non-annealed ZnS QDs and (**b**) ZnS QDs annealed at 340 °C, (**c**) SAED pattern of non-annealed ZnS QDs.

Transmission electron microscope (TEM) micrographs are shown in Fig. 2a for non-annealed ZnS and in Fig. 2b for ZnS annealed at 340 °C for 2 h. It is clear from the figure that, the nanoparticles are agglomerated, where the agglomeration is a big problem in these materials. The reason for the agglomeration is attributed to the irregular dispersion of the powder in the solution during TEM sample preparation which is also attributed to the large surface-area-to-volume ratio in NPs. This resulted in a greater number of atoms extending on the surface having unsaturated coordination and gave rise to vacant coordinate sites. This further initiates the bonding among adjacent particles which in turn led to agglomeration. Figure 2c shows the selected area electron diffraction (SAED) pattern of the non-annealed sample. The SAED consists of a set of diffused rings with spots due to the diffraction from different planes of the NPs owing to the unsystematic alignment of the NPs. All the rings that appeared in the SAED pattern are attributed to (111), (220) and (311) planes of cubic ZnS QDs NPs and this is compatible with the XRD pattern.

### FTIR studies

**Figure 3:**
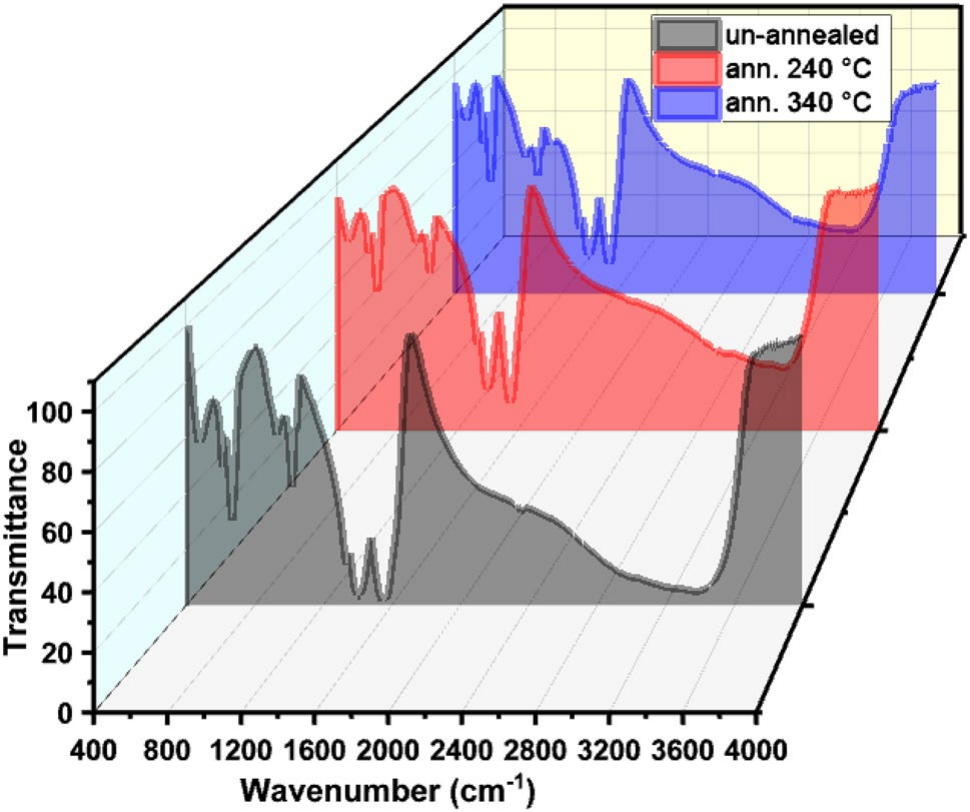
FTIR spectra of non-annealed and ZnS QDs annealed at 240 and 340 °C.

The infrared absorption peaks of functional groups present in the non-annealed and ZnS QDs annealed at 240 °C and 340 °C were studied using FTIR spectra between 400 and 4000 cm^−1^ (Fig. 3). The bands and peaks of spectral absorption observed between 670 cm ^−1^ and 480 cm^−1^ were due to stretching of Zn–S vibration modes which is consistent with previous studies^15^. The peak at 1014 cm^−1^ may be assigned to organic compounds, which may come from the alcohol used in the FTIR crucible cleaning process^23^, whereas the absorption bands with peaks at 1405 cm^−1^ and 1563 cm^−1^ which can be assigned to the symmetric stretching vibration of –COO^−^ ions. These peaks were in good agreement with previous work^24^. The high broad peak at about 3381.61 cm^−1^ can be due to the O–H stretching and H_2_O bending modes^23^.

### Photoluminescence results

**Figure 4:**
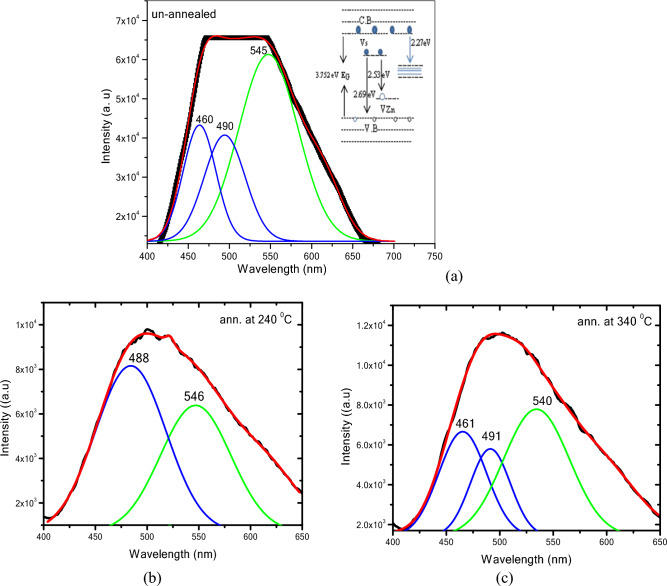
PL spectra of (**a**) non-annealed and ZnS QDs annealed at (**b**) 240 and (**c**) 340 °C.

Figure 4 shows the PL spectrum at an excitation wavelength of 360 nm for the non-annealed ZnS QDs. The PL spectrum of the non-annealed ZnS was fitted with a Gaussian function. Three emission peaks at 460, 490, and 545 nm were observed. Neither a band-to-band transition nor a peak band-edge transition could be found due to the domination of defect peaks in the finite crystalline system. The blue light emission centered at 460 nm for non-annealed and annealed at 340 °C is attributed to the defective luminescence of ZnS caused by Zn vacancy^25^. Shahid et al.^26^ attributed the emission peak observed at 460 nm to the recombination of electrons at sulfur vacancy donor levels with the holes trapped at zinc vacancy acceptor levels. The peak located at ~ 490 nm for three samples is due to Zn vacancies in ZnS QDs and this peak is also observed in another work^18^. The green emission bands observed at ~ 544 nm exhibited by all the ZnS QDs (Fig. 4a, b, c) arise from the recombination between the electrons originating from the energy level of sulfur vacancies (the donor atoms), and the holes originating from the energy level of zinc vacancies (the acceptor atoms) in the forbidden band. Similar results were observed in previous work^24^. In general, the zinc sulfide QDs contain various defects created by the synthesis conditions which are beneficial in the optical and electrical properties^25^. Due to the defects, new levels were formed above the valence band and below the conduction band and are associated with specific ZnS structure disorder due to the sulfur and zinc as simulated in the schematic diagram in Fig. 4a inset.

### Diffuse reflection (DR) spectra

**Figure 5:**
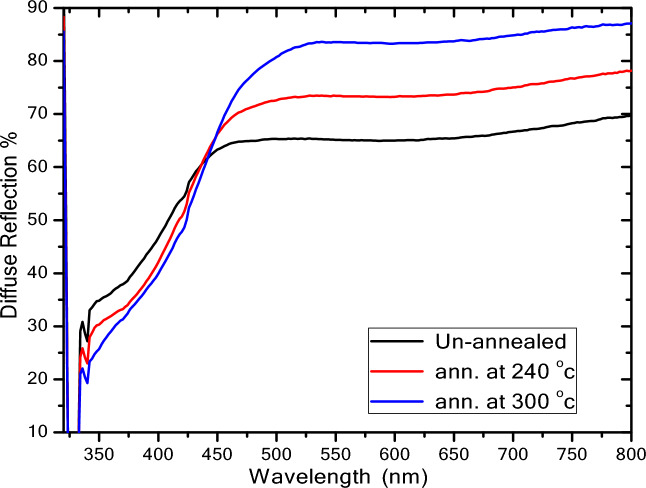
Diffuse reflection spectra of non-annealed and ZnS QDs annealed at 240 and 340 °C.

Diffuse reflection measurement by using a UV–visible spectrophotometer is used to determine the optical characteristics of ZnS QDs. The diffuse reflection spectra of ZnS QDs increased in the visible and decreased in the UV region with increasing annealing temperature (Fig. 5) may be attributed to the increase in the particle size. Also, the reflection edge was red-shifted with increasing annealing temperature, which suggested that *E*_G_ decreased with increasing temperature. Depending on the UV–vis spectrum results, *E*_G_ can be determined using the Tauc relation:

6 $$\frac{\left(\alpha h\nu \right)}{B}= {\left(h\nu -{E}_{G} \right)}^{r}$$

where *hν* is the radiated incident photon energy, *B* is a constant that depends on the transition probability and the exponential, and *α* denotes the extinction coefficient. *r* depends on the optical transition (direct or indirect) between the valence band and conduction band, where *r* = 0.5 for direct transition and *r* = 2 for indirect transition. ZnS has a direct transmission (*r* = 0.5)^15^.

The Kubelka–Munk (K–M or F(R)) method is commonly used to determine *E*_G_ by using the following equation:

7 $$F\left(R\right)= \frac{{\left(1-R\right)}^{2}}{2R}= \frac{K}{S}$$

where *R* is the % of light reflected, *K* is the absorption coefficient and *S* is the scattering coefficient. The Kubelka–Munk function can be modified and compared to Eq. (6) with the following considerations: (i) the absorption coefficient *K* = *2α* under the condition the incident radiation scatters in a perfectly diffuse manner, and (ii) considering the scattering coefficient *S* as constant concerning wavelength, so the Kubelka–Munk function can be written as^27^:

8 $${\left(F\left(R\right)\times \mathrm{h\nu }\right)}^{r}=B\left(\mathrm{h\nu }-{E}_{G}\right)$$

**Figure 6:**
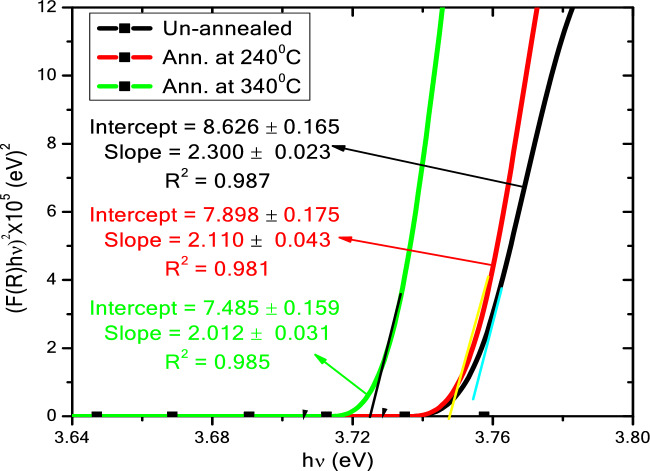
(*F(R)x hν*)^2^ versus *hν* for non-annealed and ZnS QDs annealed at 240 and 340 °C.

Figure 6 shows the curve for the plot of ${\left(F\left(R\right)\times \mathrm{h\nu }\right)}^{2}$ on the *y*-axis as a function of the incident photon energy (*hν*) on the *x*-axis for the non-annealed and annealed at 240 and 340 °C ZnS QDs. *E*_G_ can be obtained from the extrapolation on the *x*-axis. It was found that *E*_G_ were higher than that of the bulk ZnS (3.68 eV) as shown in Table 2. The increase in *E*_G_ of ZnS QDs can be explained using the quantum confinement effect.

**Table 2:** Optical band gap (*E*_G_), particles size (*P*) calculated from *E*_G_, average crystallite size (*D*) calculated by XRD, and average *a* of non-annealed and annealed ZnS QDs.

Annealed ZnS QDs	*E*_G_/eV	*P* / nm ± 0.1	Ave. *D* / nm ± 0.1	Ave. *a* / Å ± 0.01
Non-ann	3.75	4.5	4.4	5.34
Ann. at 240 °C	3.74	4.6	4.5	5.39
Ann. at 340 °C	3.72	5.1	5.6	5.35

In quantum confinement, the holes in the valence band and the electrons in the conduction band are trapped inside a surface potential barrier. Because the electrons and holes are confined, the least energy optical transition from the valence to the conduction band will increase in energy, thereby increasing the band gap^28^:

9 $${E}_{G}-{E}_{g}^{bulk}=\frac{{h}^{2}{\pi }^{2}}{{2\mu P}^{2}}-\frac{1.786{e}^{2}}{{\varepsilon }_{r}}+0.284{E}_{r}$$

where ${E}_{g}^{bulk}$ is the band gap of the corresponding bulk material, *P* is the particles size, ${\varepsilon }_{r}$ is the dielectric constant of the bulk and equal to 8.76 for ZnS, μ is the reduced mass of the exciton given by $\frac{{m}_{e}^{*}{m}_{h}^{*}}{{m}_{e}^{*}+{m}_{h}^{*}}$, with ${m}_{e}^{*}=0.34 {m}_{o}$, ${m}_{h}^{*}=0.23{ m}_{o}$, where, ${m}_{e}^{*}$ and ${m}_{h}^{*}$ effective mass of electron and hole, respectively. *m*_o_ = 9.1 × 10^–31^ kg, and *E*_*r*_ is the Rydberg energy. Substituting μ, ${\varepsilon }_{r}$ and neglecting the polarization term ($0.284{E}_{r}$) Eq. (9) can be written as:

10 $${E}_{G}-{E}_{g}^{bulk}= \frac{2.7442}{{P}^{2}}+\frac{0.2963}{P}$$

By using Eq. (10) and the values of the *E*_G_, the particles size (*P*) of non-annealed and annealed ZnS were calculated and summarized in Table 2.

**Figure 7:**
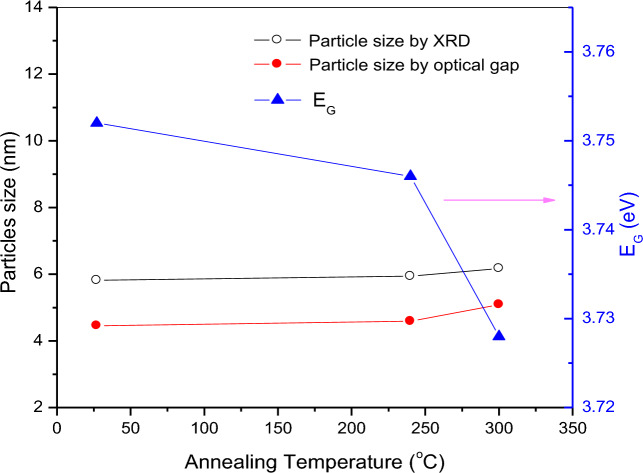
The optical band gap (*E*_G_) and particles size (*P*) calculated from *E*_G_ for non-annealed and annealed ZnS QDs.

Figure 7 shows *E*_G_, *P* and *D* of ZnS QDs as a function of annealing temperature. This figure illustrates two methods (*P* and *D*) to calculate the dot size of ZnS QDs. Both methods showed that the dot size of ZnS QDs increased with increasing annealing temperature. However, *E*_G_ decreased with increasing annealing temperature. The decrease in *E*_G_ may be attributed to the increase in particles size and decrease in the strain^29^. The data of *E*_G_ and *D* presented in this study were compared to the previous works with various synthesis methods as shown in Table 3^25,30–36^. Generally, the particles size, structural and optical properties of ZnS nanopowders were found to be sensitively dependent on the annealing temperature. Similar variations in *E*_G_ was also observed on other nanoparticle systems such as ZnSe thin films^29^, ZnO nanorods^37^ and ZnS doped Au, Mn and Ga^38^.

**Table 3:** Average crystallite size (*D*), optical band gap (*E*_G_), and particles size (*P*) for the non-annealed and annealed ZnS with various synthesis method.

Annealed ZnS QDs	Synthesis Method	*D* (nm)	*E*_G_ (eV)	Ref
Non-Ann	Co-precipitation method—without capping agent	4.4 ± 0.1	3.75	This work
240 °C		4.5 ± 0.1	3.74	
340 °C		5.6 ± 0.1	3.72	
950 °C	Solid-state reaction—2 h under a pressure of 1 × 10^−3^ mbar—Thin Film	20 ± 0.1	3.52	^31^
Non-Ann	Co-precipitation—capped with 2-mercaptoethonal	2.66 ± 0.01	4	^32^
500 °C		34.60 ± 0.01	< 4	
Non-Ann	Refluxing technique at 80 °C capped with poly vinyl pyrrolidone (PVP)	5–8 ± 0.1	3.93	^30^
400 °C	Solid-state reaction method-capped with thiourea—for 4 h in nitrogen atmosphere	2.641 ± 0.001	3.83	^33^
Non-Ann	Co-precipitation in aqueous medium	3.1 ± 0.1	4.14	^34^
Non-Ann	Wet chemical method—different concentration of source of Zn (ZnCl_2_)-without capping agent	3.0 ± 0.1	4.46	^25^
		3.5 ± 0.1	4.06	
		4.5 ± 0.1	3.55	
Non-Ann	Chemical precipitation method-dried in hot air oven at 80 °C for 2 h	4 ± 0.1	4.14	^35^
Non-Ann	Chemical precipitation method	2.5 ± 0.1	5.14	^36^
200 °C		4.3 ± 0.1	–	
400 °C		43.7 ± 0.1	–	
600 °C		49 ± 0.1	3.82	

## Conclusions

The effects of different annealing temperatures on the structural and optical properties of ZnS QDs are reported in this paper. FTIR and XRD confirmed the formation of zinc sulfide. Compared to the annealed samples, the non-annealed ZnS QDs have a smaller average crystallite size (4.4 nm) and larger band gap (3.75 eV). The average crystallite size of ZnS QDs increased from 4.5 to 5.6 nm while the band gap decreased from 3.74 to 3.72 eV for annealing temperatures of 240 and 340 °C, respectively. A red shift in UV–Visible spectra was observed when the annealing temperature was increased. PL spectra showed the presence of defects within the sample which may be useful in the electrical and optical properties. This work showed that annealing temperature is useful for controlling the dot size and *E*_G_ of ZnS QDs. Further studies with higher annealing temperatures and longer times can be performed to investigate their effects on the ZnS QDs properties.

## Data Availability

The datasets used and/or analyzed during the current study are available from the corresponding author upon reasonable request.
